# Metabolomics analyses in non-diabetic middle-aged individuals reveal metabolites impacting early glucose disturbances and insulin sensitivity

**DOI:** 10.1007/s11306-020-01653-7

**Published:** 2020-03-02

**Authors:** Maxime M. Bos, Raymond Noordam, Kate Bennett, Marian Beekman, Dennis O. Mook-Kanamori, Ko Willems van Dijk, P. Eline Slagboom, Torbjörn Lundstedt, Izabella Surowiec, Diana van Heemst

**Affiliations:** 1 Section of Gerontology and Geriatrics, Department of Internal Medicine, Leiden University Medical Center, PO Box 9600, 2300 RC Leiden, The Netherlands; 2 AcureOmics AB, Umeå, Sweden; 3 Section of Molecular Epidemiology, Department of Biomedical Data Sciences, Leiden University Medical Center, Leiden, The Netherlands; 4 Department of Clinical Epidemiology, Leiden University Medical Center, Leiden, The Netherlands; 5 Department of Public Health and Primary Care, Leiden University Medical Center, Leiden, The Netherlands; 6 Department of Human Genetics, Leiden University Medical Center, Leiden, The Netherlands; 7 Division of Endocrinology, Department of Internal Medicine, Leiden University Medical Center, Leiden, The Netherlands; 8 Leiden Laboratory for Experimental Vascular Medicine, Leiden University Medical Center, Leiden, The Netherlands; 9 Department of Chemistry, Umeå University, Umeå, Sweden

**Keywords:** Metabolomics, Type 2 diabetes mellitus, Glucose metabolism, Insulin sensitivity

## Abstract

**Introduction:**

Several plasma metabolites have been associated with insulin resistance and type 2 diabetes mellitus.

**Objectives:**

We aimed to identify plasma metabolites associated with different indices of early disturbances in glucose metabolism and insulin sensitivity.

**Methods:**

This cross-sectional study was conducted in a subsample of the Leiden Longevity Study comprising individuals without a history of diabetes mellitus (n = 233) with a mean age of 63.3 ± 6.7 years of which 48.1% were men. We tested for associations of fasting glucose, fasting insulin, HOMA-IR, Matsuda Index, Insulinogenic Index and glycated hemoglobin with metabolites (Swedish Metabolomics Platform) using linear regression analysis adjusted for age, sex and BMI. Results were validated internally using an independent metabolomics platform (Biocrates platform) and replicated externally in the independent Netherlands Epidemiology of Obesity (NEO) study (Metabolon platform) (n = 545, mean age of 55.8 ± 6.0 years of which 48.6% were men). Moreover, in the NEO study, we replicated our analyses in individuals with diabetes mellitus (cases: n = 36; controls = 561).

**Results:**

Out of the 34 metabolites, a total of 12 plasma metabolites were associated with different indices of disturbances in glucose metabolism and insulin sensitivity in individuals without diabetes mellitus. These findings were validated using a different metabolomics platform as well as in an independent cohort of non-diabetics. Moreover, tyrosine, alanine, valine, tryptophan and alpha-ketoglutaric acid levels were higher in individuals with diabetes mellitus.

**Conclusion:**

We found several plasma metabolites that are associated with early disturbances in glucose metabolism and insulin sensitivity of which five were also higher in individuals with diabetes mellitus.

**Electronic supplementary material:**

The online version of this article (10.1007/s11306-020-01653-7) contains supplementary material, which is available to authorized users.

## Introduction

Over the past decades, the incidence of type 2 diabetes mellitus (T2D) has increased, partly due to the ever increasing prevalence of obesity (Ogden et al. 2014a, b). T2D is preceded by several disturbances in glucose metabolism, which can be recognized in the pre-disease state. A novel approach that is being increasingly used to gain additional insight in the disturbances in glucose metabolism before the development of T2D is high-throughput metabolomics. Metabolomics offers the possibility to comprehensively measure a broad range of metabolites in tissues and biological fluids. Multiple observational and causal association studies revealed metabolites such as phospholipids, triacylglycerols, ketone bodies, sphingomyelins, acyl-carnitines and organic acids to be linked to future risk of T2D onset (Mahendran et al. 2013a, b; Tillin et al. 2015; Wurtz et al. 2012, 2013; Mook-Kanamori et al. 2014; t Hart et al. 2018; Guasch-Ferre et al. 2016; Liu et al. 2017; Wang et al. 2017; Fall et al. 2016).

To recognize early stages of T2D one needs increased understanding of the mechanisms contributing to disturbances in glucose metabolism and insulin resistance among individuals without T2D. To quantify early disturbances in glucose metabolism, several indices have been developed based on an oral glucose tolerance test or based on fasting/postprandial plasma and insulin levels. For example, the homeostatic model assessments can be used to quantify insulin resistance (HOMA-IR) based on fasting glucose and insulin levels (Retnakaran et al. 2008; Singh and Saxena, 2010; Matthews et al. 1985; Wallace et al. 2004). If both fasting and post-prandial measures are available, indices such as the Matsuda Index or Insulinogenic index can be used to assess insulin resistance and β-cell function respectively^15^. The Matsuda Index reflects both hepatic and peripheral tissue insulin sensitivity and is therefore considered to be an index of whole-body insulin sensitivity (Retnakaran et al. 2008; Singh and Saxena, 2010). These and other indices all reflect different yet partly overlapping aspects of glucose tolerance and insulin sensitivity that play a role in T2D onset.

In this study, we investigated which metabolites measured by a GC–MS assay are associated with early indices of disturbances in glucose metabolism and insulin sensitivity in individuals that do not use glucose lowering drugs and do not have a history of diabetes mellitus. To this end, we performed association analyses of plasma metabolites with fasting glucose, fasting insulin, the insulinogenic index, two indices of insulin resistance (HOMA-IR and Matsuda) and HbA1c. Subsequently, we replicated the significant findings internally using a different metabolomics platform as well as in an independent cohort in both non-diabetics and individuals with diabetes mellitus.

## Methods

### Study design of the Leiden Longevity Study

The main analyses for the present study were embedded in the Leiden Longevity Study, which aims to investigate biomarkers associated with familial longevity and healthy ageing. A more detailed description of the study design and recruitment strategy has been described elsewhere (Schoenmaker et al. 2006). In short, between 2003 and 2006, a total of 421 long-lived families were recruited, without selection based on health condition or demographics. Families were included when at least two long-lived siblings were still alive and fulfilled the age criteria of being at least 89 years for men and 91 years for women. Of these long-lived families, we also recruited 1671 of their offspring and 744 partners thereof as controls resembling the general Dutch population at middle age.

For the present study, we used fasting and postprandial blood samples collected between 2006 and 2008 from a subpopulation (N = 280) of the Leiden Longevity Study who lived in close approximation (< 45 min by car) from the research center, as described previously (Rozing et al. 2010). Glucose tolerance was assessed according to a 2‐h oral glucose tolerance test, conducted with a standard loading dose of 75 g of glucose per 300 mL of water and venous blood samples were drawn at time points of 0, 30, 60, and 120 min after glucose loading. We excluded participants that used glucose lowering drugs, had a history of diabetes mellitus, who were not fasted before taking glucose tolerance test, had no fasting glucose or insulin measures, or had incomplete/unreliable postprandial data. In the present study, we therefore included a total of 233 participants.

The Leiden Longevity Study was approved by the medical ethics committee of the Leiden University Medical Center. All participants provided written informed consent.

### Measures of insulin resistance

Fasting plasma glucose concentrations and glycated hemoglobin levels were determined by enzymatic and colorimetric methods (Roche Modular Analytics P800, Roche Diagnostics, Mannheim, Germany; CV < 5%) and serum insulin concentrations were determined by an immunometric method (Siemens Immulite 2500, Siemens Healthcare Diagnostics, Breda, The Netherlands; CV < 5%). All analyses were performed in the central clinical chemical laboratory of the Leiden University Medical Center. Fasting glucose and insulin levels were used to calculate the Homeostatic Model Assessment of Insulin Resistance (HOMA-IR) index as a marker for hepatic insulin resistance. The HOMA-IR was calculated using (fasting insulin * fasting glucose)/22.5. The Insulinogenic Index was calculated as (insulin concentration_30min_ − fasting insulin concentration)/(glucose concentration_30min_ − fasting glucose concentration) and the Matsuda Index as 1000/(squared root (fasting glucose * fasting insulin) * (average glucose * average insulin)) (Singh and Saxena, 2010).

### Metabolomic profiling

Metabolomic profiling has been performed in three separate analytical batches analysed on three different days, of which the first batch consisted of cases with different levels of metabolic syndrome score as described previously (Surowiec et al. 2019). The samples of the other participants were separated based on the gender of the participants, resulting in a batch of only women (Batch 2) or only men (Batch 3). These three batches were used for the analyses in this study. Fasting plasma samples from the participants were thawed on ice; 630 µL of extraction mixture (H_2_O:methanol (1:9, v/v)) was added to 70 µL of plasma. Extraction of the metabolites from the sample was then carried out using a MM301 vibration Mill (Retsch GmbH & Co. KG, Haan, Germany) at a frequency of 30 Hz for 2 min. Samples were stored on ice for 2 h to allow protein precipitation, after which they were centrifuged at a relative central force of 18,000 for 10 min at 4 °C. An aliquot (200 µL) of the resulting supernatant was transferred to a glass vial and evaporated to dryness at room temperature in a miVac QUATTRO concentrator (Genevac LTD, Ipswich, UK). Gas chromatography-mass spectrometry (GC–MS) (Batch 1, 2 and 3) analyses was performed after metabolite derivatization as described before (A et al. 2005).

Non-processed files from GC–MS were exported in NetCDF format to a MATLAB-based in-house script where all data pre-treatment procedures such as baseline correction, chromatogram alignment, and peak deconvolution were performed. Metabolite identification, was implemented within the script and was based on the retention index (RI) values and MS spectra from the in-house mass spectra library established by the Swedish Metabolomics Centre (Umeå, Sweden) and consisting of 585 compounds (Level 1 identification according to the Metabolomics Standards Initiative (Salek et al. 2013)). However, since the library covers a wide range of compound classes, and includes a significant number of compounds present in samples from other species than humans (for example plants, bacteria, etc.), as expected, not all metabolites present in the library could be identified in our samples. In the three analytical batches, respectively 105, 48 and 57 compounds were identified, with 34 compounds present in all three batches. In order to strengthen our findings, in calculations we only included those metabolites that were confidently measured in all three batches.

### Internal validation

For internal validation, we used metabolomic data measured in the Leiden Longevity Study using the commercially available AbsoluteIDQ p180 (Biocrates Life Sciences, AG, Innsbruck, Austria) mass spectrometry (MS)-based assay kit. Fasting plasma samples were collected from study participants and were stored at − 80 °C. A different (unique) aliquot of the sample was used, thus avoiding potential bias caused by introducing a freeze/thaw step when using the same sample for discovery and validation. In this study, we included only those metabolites that overlapped with those that were identified using the GC–MS approach as being significantly associated with indices of disturbances in glucose metabolism and insulin sensitivity after correction for multiple testing, note that only 5 out of the 12 significant metabolites were also present on the Biocrates AbsoluteIDQ p180 kit, namely: phenylalanine, proline, tryptophan, tyrosine and valine.

### External validation

For external replication, we used data from the independent Netherlands Epidemiology of Obesity (NEO) study, which had no overlap with the LLS study in regard of study participants. A detailed description of the NEO study design and rationale has been provided elsewhere (de Mutsert et al. 2013). We included participants with available data on the same indices of glucose metabolism (e.g. fasting glucose and insulin, HOMA-IR, Matsuda Index, Insulinogenic Index and glycated hemoglobin) as available in the LLS and an LC–MS and GC–MS based metabolic profile (Metabolon, Inc.), who were fasted and drank a complete liquid mixed meal (n = 545). Moreover, we assessed the associations in participants with diabetes mellitus (cases: n = 36; controls = 561). Presence of diabetes mellitus was defined as the usage of glucose lowering medication and/or having a history of diabetes mellitus and/or having fasting glucose levels of ≥ 7.0 mmol/L.

Metabolomic profiling was performed using ultrahigh-performance liquid-phase chromatography and gas chromatography separation, coupled with tandem mass spectrometry at Metabolon, Inc. using established procedures (12). Fasting plasma samples were collected from study participants and were stored at − 80 °C. Here, we only analyzed the identified metabolites in the LLS association study (those metabolites that were significant after correction for multiple testing) and did not include all of the metabolites measured using this platform.

### Statistical analyses

All analyses were performed using the R statistical environment (Team 2008). Before analyses, we removed those observations that could be considered outliers based on biological plausible reference values for these measurements. To gain insight in the interrelations of the metabolites included in the present study, we estimated a correlation network using Pearson correlations for the 34 scaled metabolites measured using the GC–MS approach in the LLS study. The Pearson correlations for each batch were obtained separately and we took the average correlation of these batches taking into account the number of individuals included in each batch. The qgraph package (Epskamp et al. 2012), using an absolute weight of edges of > 0.5 to be shown, was used to obtain the plot.

We conducted univariate analyses on the metabolites using linear regression analysis adjusted for age, sex and body mass index. All measures of glucose metabolism were log-transformed, independent of normality of the variables in order to improve comparability among the measurements. Peak areas (GC–MS) and metabolite concentrations (Biocrates LC–MS platform) were log-transformed and subsequently standardized using scaling to approach a standard normal distribution (mean = 0, s.d. = 1). Hence, results can be interpreted as the difference in standard deviation per unit increase in measured index. Because of the applied distribution of the individuals in the different batches rather than random allocation, proper correction for batch effect was not possible. Instead, we conducted all univariate analyses separately for the different batches; derived effect estimates were subsequently meta-analysed using a fixed-effect model, assuming a similar direction of effect among batches, by using the rmeta package (Lumley 2018). Meta-analysis was only performed for those metabolites that were measured in all three batches, which resulted in a total of 34 metabolites. To correct for multiple testing, we calculated the number of independent metabolites based on the methodology described by Li and Ji (2005), and corrected our threshold for statistical significance accordingly. We obtained a total of 24 independent metabolites and used a p-value of 0.05/24 (~ 0.0021) as a threshold for significance. Analyses were visualized using the ggplot2 package (Wickham 2009).

For internal validation, we repeated our analyses using significant metabolites from our discovery analyses. Of these metabolites, only five metabolites were overlapping with the Biocrates AbsoluteIDQ p180 kit. We performed the same analyses using the same statistics and covariables as in the discovery analyses, however, we used a p-value of < 0.05 as level of statistical significance. For external replication, analyses were repeated with the metabolites that were identified in the LLS study and could also be measured by the GC–MS Metabolon platform, comprising twelve metabolites present in the NEO study and Metabolon data. We used the same statistical methods as described previously, however, since we tested hypothesis generated in the discovery phase, we considered a two-sided p-value of < 0.05 as statistically significant.

## Results

### Population characteristics

A total of 233 participants were included in the main study with a mean age of 63.3 ± 6.7 years of which 48.1% were men. The population characteristics of this study are shown in Table 1, separately for each batch and combined for all batches. Characteristics of the study population are presented as means with accompanying standard deviations for normally distributed variables, median with interquartile range for non-normal distributed variables and number with percentages for proportional variables. Participants in Batch 3 used slightly more antihypertensive and lipid lowering medication as compared to the other batches.

**Table 1:** Characteristics of study population stratified by batches and combined

	Batch 1 (N = 82)	Batch 2 (N = 78)	Batch 3 (N = 73)	Combined (N = 233)
Age in years, mean (SD)	63.2 (6.2)	61.5 (7.0)	65.4 (6.4)	63.3 (6.7)
Men, N (%)	39 (47.6)	0 (0.0)	73 (100.0)	112 (48.1)
BMI in kg/m^2^, mean (SD)	26.3 (4.9)	25.9 (3.2)	26.9 (3.0)	26.5 (3.8)
Body fat %, mean (SD)	31.3 (9.4)	35.6 (6.5)	26.3 (5.6)	31.2 (8.3)
Fasting glucose in mmol/L, mean (SD)	5.2 (0.7)	5 (0.4)	5.1 (0.5)	5.1 (0.5)
Fasting insulin in mU/L, median (IQR)	7.0 (4.0–11.8)	6.0 (4.0–9.0)	7.0 (4.0–10.0)	6.0 (4.0–10.0)
HOMA-IR, median (IQR)	1.6 (0.8–2.7)	1.2 (0.9–2.0)	1.5 (1.0–2.2)	1.5 (0.9–2.2)
Insulinogenic Index, median (IQR)	0.6 (0.4–1.1)	0.8 (0.5–1.2)	0.7 (0.4–1.2)	0.7 (0.4–1.1)
Matsuda Index, median (IQR)	24.6 (12.7–45.3)	26.9 (18.7–42.7)	25.1 (17.4–35.7)	26.3 (17.1–41.2)
HbA1c in %, mean (SD)	5.1 (0.4)	5.1 (0.4)	5.0 (0.5)	5.1 (0.4)
ASAT in U/L, median (IQR)	22.1 (7.2)	22.5 (8.6)	21.5 (4.6)	22.1 (7.0)
ALAT in U/L, median (IQR)	16.0 (13.0–19.8)	15.0 (12.0–19.8)	17.0 (15.0–21.0)	16.0 (13.0–20.0)
GGT in U/L, median (IQR)	22.0 (15.0–35.0)	18.0 (13.0–24.8)	29.0 (21.0–40.0)	22.0 (15.0–33.0)
hsCRP in mg/dL, median (IQR)	1.2 (0.7–2.5)	1.1 (0.7–1.9)	1.0 (0.6–2.0)	1.1 (0.6–2.3)
Total cholesterol in mmol/L, mean (SD)	5.3 (1.1)	5.6 (1.0)	5.1 (1.0)	5.3 (1.0)
HDL-cholesterol in mmol/L, mean (SD)	1.5 (0.4)	1.7 (0.5)	1.4 (0.4)	1.5 (0.4)
LDL-cholesterol in mmol/L, mean (SD)	3.2 (0.9)	3.3 (0.9)	3.1 (0.9)	3.2 (0.9)
Triglycerides in mmol/L, mean (SD)	1.5 (0.8)	1.3 (0.5)	1.3 (0.5)	1.4 (0.6)
Hypertension, N (%)	20 (24.4)	19 (24.4)	25 (34.2)	64 (27.5)
Statin use, N (%)	7 (8.50	10 (12.8)	10 (13.7)	27 (11.6)

*BMI* body mass index, *ALAT* alanine transaminase, *ASAT* aspartate transaminase, *GGT* gamma-glutamyltransferase, *HbA1c* glycated haemoglobin, *HDL* high-density lipoprotein, *HOMA-IR* Homeostatic Model Assessment of Insulin Resistance, *hsCRP* high-sensitivity C-reactive protein, *IQR* interquartile range, *LDL* low-density lipoprotein, *N* number of participants, *SD* standard deviation

### Network analysis of metabolites

**Fig. 1:**
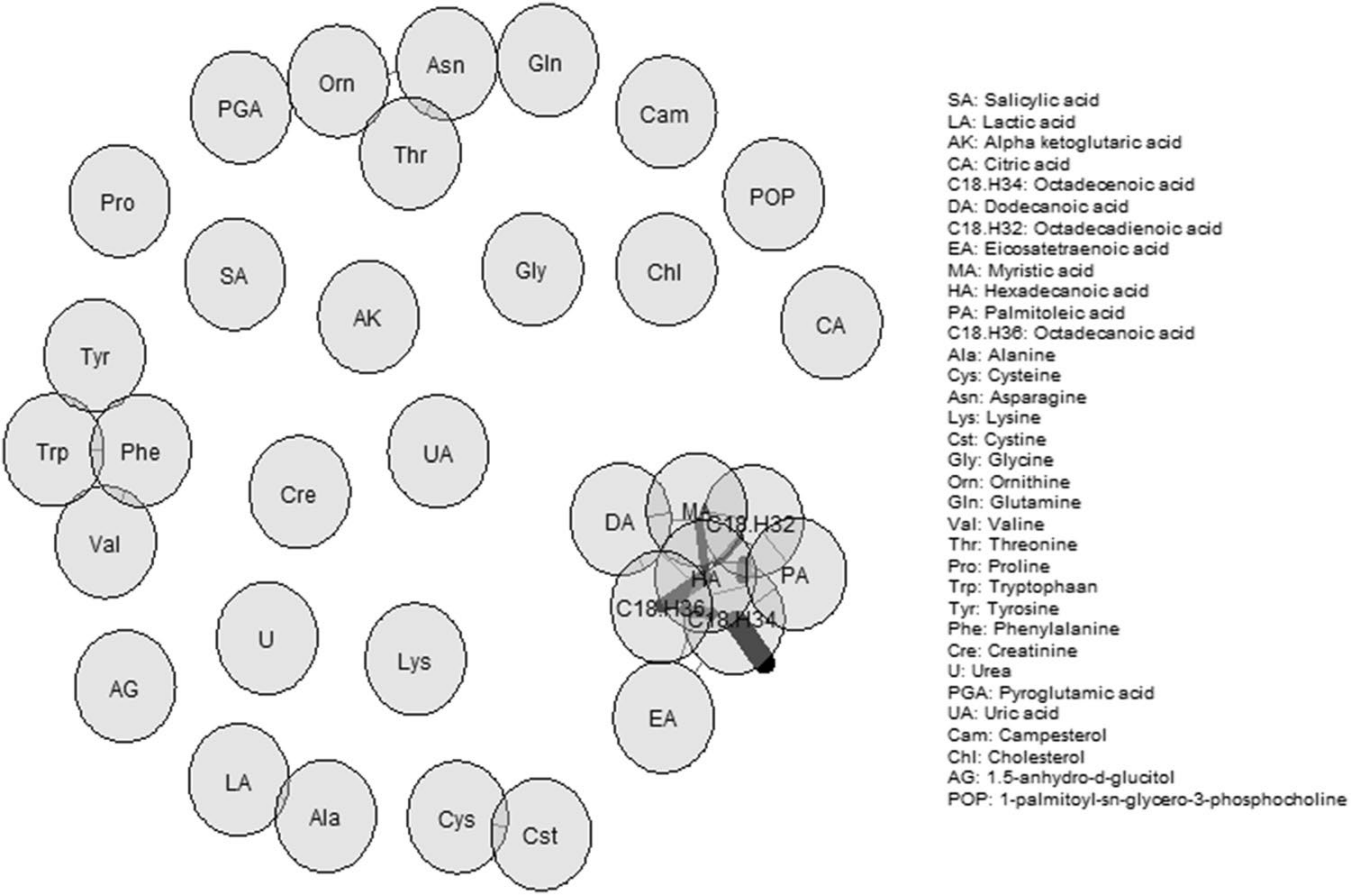
Correlation network estimated using Pearson correlations. Each node represents one metabolite. The edges indicate the strength of the correlation. For this plot, only edges with an absolute weight of > 0.5 are shown

In order to have a better understanding of the interrelations of metabolites in this study, we calculated and visualized the correlations between the different metabolites. In Fig. 1, a correlation network is shown of the primary metabolite set tested in the LLS study comprising 34 metabolites. Several amino acids were correlated, such as tryptophan, tyrosine, phenylalanine and valine. The strongest correlations were observed between fatty acids, such as myristic acid, dodecanoic acid and octadecanoic acid.

### Associations between different indices of glucose metabolism with the metabolomic profile

**Fig. 2:**
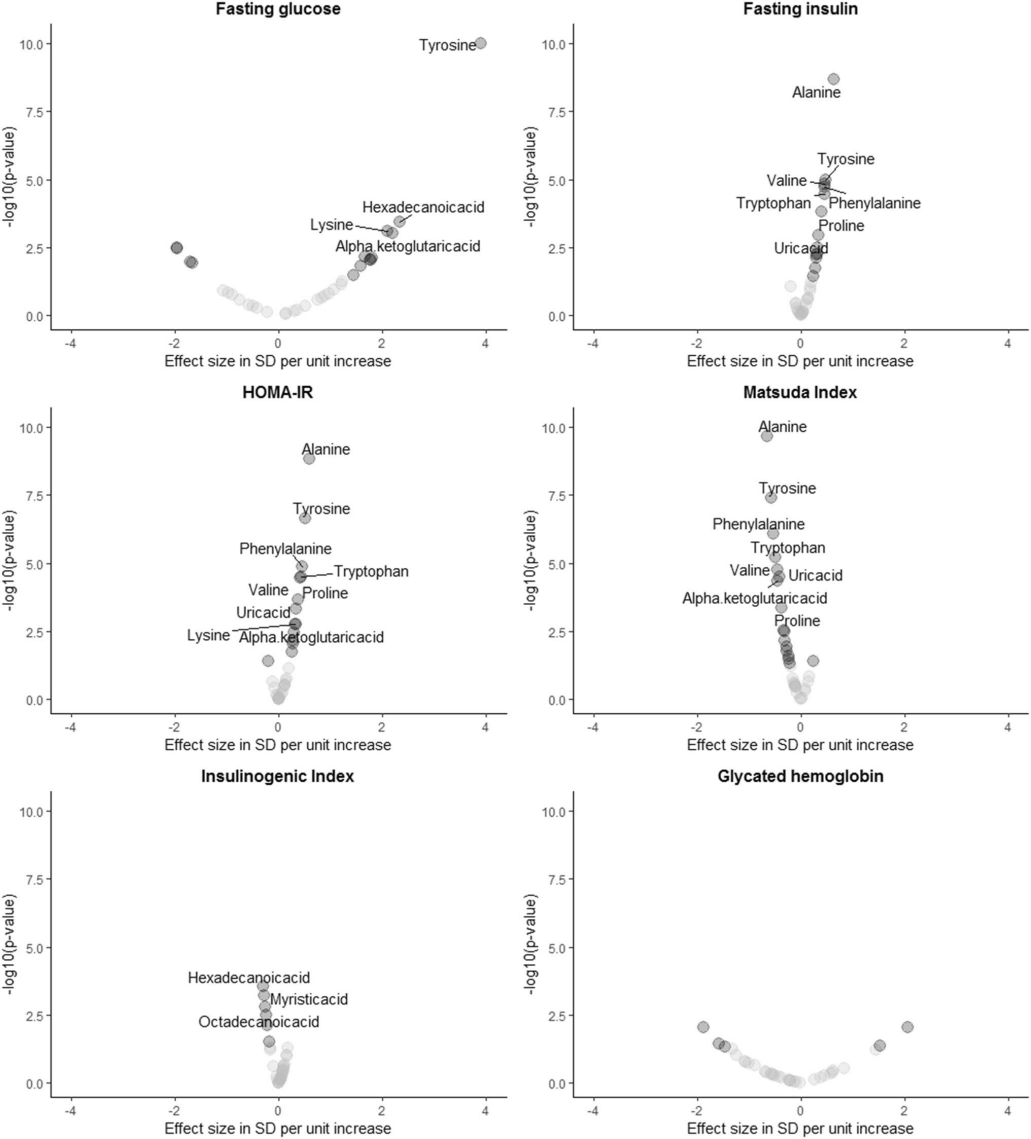
Associations of plasma metabolites with measures of glucose metabolism. Analyses can be interpreted as the difference in metabolite level in standard deviation in relation to unit increase of the exposure. The difference in exposure (in standard deviation) is presented on the x-axis; the -log(p-value) of the comparison is presented on the y-axis. Metabolites that were labelled in the figures were those that remained significant after correction for multiple testing (p < 2.1e−3); compounds with a p-value < 0.05 are presented as solid black dots

We first tested for association of the 34 single metabolites that were measured in all three batches with six variables of glucose metabolism and insulin sensitivity in the Leiden Longevity Study adjusted for age, sex and BMI. Twelve metabolites were identified to be associated (p-value < 0.05/24) with markers of glucose metabolism, as shown in Fig. 2 and Supplementary Table 1. Tyrosine, hexadecanoic acid, lysine and alpha-ketoglutaric acid levels were associated with fasting glucose concentrations. Levels of alanine, tyrosine, valine, phenylalanine, tryptophan, proline and uric acid were positively associated with fasting insulin and HOMA-IR and negatively associated with the Matsuda index. Alpha-ketoglutaric acid was positively associated with HOMA-IR and negatively with the Matsuda Index, however, not with fasting insulin. Moreover, lysine levels were positively associated with HOMA-IR, however, not with fasting insulin concentrations or the Matsuda Index. Levels of hexadecanoic acid, myristic acid and octadecanoic acid were associated with Insulinogenic Index. None of these metabolites were associated with either fasting insulin, HOMA-IR or the Matsuda Index. However, hexadecanoic acid levels positively associated with fasting glucose concentrations. Finally, glycated hemoglobin was not associated with any of the metabolites.

### Internal validation of main results

Next, we validated the significant observations using the Biocrates platform in order for us to test the robustness of these findings when using a different metabolomics platform. Note that only 5 out of the 12 significant metabolites overlapped with the Biocrates platform (phenylalanine, proline, tryptophan, tyrosine and valine). In Supplementary Table 2, effect estimates for the association of measures of glucose metabolism with these metabolites are shown. Effect estimates pointed in the same direction and were similar to those observed using the Swedish Metabolomics Platform. For fasting glucose, only tyrosine was present in the Biocrates platform and we were able to validate this result. Proline, tryptophan, tyrosine and valine were positively associated with fasting insulin, HOMA-IR and negatively with the Matsuda Index, thereby validating the previous results. Phenylalanine negatively associated with the Matsuda Index, however, not with fasting insulin and the HOMA-IR, thereby only validating the result for the Matsuda Index. The metabolites that associated with the Insulinogenic Index were not present in the Biocrates platform and therefore, we did not validate these findings. Moreover, for glycated hemoglobin we did not identify any significant associations and therefore we did not validate any finding.

### External validation of main results in individuals with and without diabetes mellitus

We validated our results for the identified metabolites in an independent subset of the NEO cohort in individuals without diagnosis of diabetes mellitus that did not use glucose lowering medication using the same indices of glucose metabolism and insulin sensitivity. As can be seen in Tables 2 and 3, tyrosine, hexadecanoic acid and alpha-ketoglutaric acid were positively associated with levels of fasting glucose, thereby replicating the associations as observed in the LLS study, except for lysine. Levels of tyrosine, alanine, valine and phenylalanine were positively associated with fasting insulin and HOMA-IR levels in both the LLS and the NEO study. Additionally, alpha-ketoglutaric acid significantly associated with HOMA-IR. However, proline, tryptophan (fasting insulin and HOMA-IR) and uric acid (HOMA-IR) did not associate in the NEO study. Tyrosine, alanine, valine, phenylalanine, alpha-ketoglutaric acid, hexadecanoic acid and uric acid were negatively associated with the Matsuda Index, similar as in the LLS study. However, tryptophan was not associated with the Matsuda Index in the NEO study. None of the metabolites that associated with the Insulinogenic Index in the LLS study were replicated in the NEO study.

**Table 2:** Associations of blood metabolites with measures of glucose metabolism in NEO using Metabolon

	Fasting glucose			Fasting insulin			HOMA-IR			Matsuda Index			Insulinogenic Index		
	Beta	SE	P-value	Beta	SE	P-value	Beta	SE	P-value	Beta	SE	P-value	Beta	SE	p-value
Tyrosine	1.29	0.34	< 0.001	0.26	0.07	< 0.001	0.27	0.07	< 0.001	− 0.26	0.08	0.001			
Lysine	− 0.45	0.36	0.211				− 0.11	0.07	0.122						
Alanine				0.36	0.07	< 0.001	0.37	0.07	< 0.001	− 0.48	0.08	< 0.001			
Valine				0.14	0.06	0.025	0.15	0.06	0.015	− 0.22	0.07	0.002			
Proline				0.14	0.07	0.054	0.11	0.07	0.092	− 0.09	0.08	0.239			
Phenylalanine				0.30	0.07	< 0.001	0.27	0.07	< 0.001	− 0.35	0.08	< 0.001			
Tryptophan				0.13	0.07	0.079	0.11	0.07	0.111	− 0.12	0.08	0.118			
Hexadecanoic acid	0.84	0.36	0.021										− 0.07	0.05	0.123
Alpha-ketoglutaric acid	1.38	0.35	< 0.001				0.45	0.07	< 0.001	− 0.54	0.08	< 0.001			
Myristic acid													− 0.04	0.05	0.431
Octadecanoic acid													− 0.04	0.05	0.354
Uric acid							0.09	0.06	0.148	− 0.16	0.07	0.027			

Data presented as beta’s with accompanying standard errors (SE) and p-values. Results can be interpreted as the difference in metabolite level in standard deviation in relation to unit increase of the glycemic trait. Analyses are adjusted for age, sex and body mass index

In addition, we studied which of the twelve metabolites associated with diabetes mellitus (cases = 36; controls = 561). As compared to individuals without diabetes mellitus, individuals with diabetes mellitus had higher levels of tyrosine, alanine, valine, tryptophan and alpha-ketoglutaric acid (Table 3).

**Table 3:** Associations of blood metabolites with measures of glucose metabolism in NEO using Metabolon

	Diabetes Mellitus		
	Beta	SE	p-value
Tyrosine	1.93	(1.58–2.28)	< 0.001
Lysine	1.31	(0.96–1.66)	0.134
Alanine	1.59	(1.24–1.94)	0.010
Valine	1.94	(1.59–2.29)	< 0.001
Proline	1.30	(0.94–1.65)	0.150
Phenylalanine	1.35	(1.00–1.70)	0.097
Tryptophan	1.47	(1.12–1.82	0.033
Hexadecanoic acid	0.98	(0.63–1.33)	0.902
Alpha-ketoglutaric acid	1.71	(1.36–2.06)	0.003
Myristic acid	1.16	(0.81 – 1.51)	0.409
Octadecanoic acid	0.84	(0.48 – 1.19)	0.319
Uric acid	1.14	(0.78 – 1.49)	0.476

Data presented as odd ratio’s (OR) with accompanying 95% confidence interval (95%CI) and p-values. Analyses are adjusted for age, sex and body mass index

## Discussion

We identified twelve metabolites to be associated with different indices of glucose metabolism and insulin sensitivity, in individuals without diabetes mellitus from the general population. These results were largely validated and externally replicated in an independent cohort. Moreover, five of the twelve metabolites (tyrosine, alanine, valine, tryptophan and alpha-ketoglutaric acid) were associated with diabetes mellitus. These results indicate that specific early alterations in the metabolic profile are already present in individuals without diabetes mellitus and these findings may therefore improve the understanding of mechanisms involved in diabetes mellitus etiology.

To date, several amino acids, sugar metabolites, and lipids have been associated with T2D risk in observational studies and causality has been investigated using Mendelian Randomization (Mahendran et al. 2013a, b; Tillin et al. 2015; Wurtz et al. 2012, 2013; Mook-Kanamori et al. 2014; t Hart et al. 2018; Guasch-Ferre et al. 2016; Liu et al. 2017; Wang et al. 2017; Fall et al. 2016; Rawat et al. 2019; Newsholme et al. 2005). The present study replicates these observational findings in participants without diabetes mellitus, indicating that alterations in plasma metabolites levels in relation to perturbed glucose metabolism are already present in the non-diabetic population. For example, alanine, valine, tyrosine and phenylalanine have been consistently associated with the risk of developing type 2 diabetes mellitus in different studies (Guasch-Ferre et al. 2016). A better understanding of the molecular mechanisms by which amino acids may impact insulin resistance may aid the identification of novel targets for future diabetes therapies. One of these pathways is via mitochondrial metabolism and the exocytosis of insulin granules. Next to ATP, the main factor in insulin secretion, other factors such as nucleotides, amino acids, enzymes or transporters and alanine aminotransferase have been identified to mediate insulin secretion. Alanine, our strongest association, has previously been described to directly affect β-cell function and insulin secretion (Newsholme et al. ). In line, in this study we observed these metabolites to be associated with insulin sensitivity in individuals without diabetes mellitus, as well as in individuals with diabetes mellitus.

Some of the identified associations can be explained by correlations between the metabolites. For example, tyrosine, valine and phenylalanine were correlated which were also observed together in the analyses addressing the association with fasting insulin, HOMA-IR and the Matsuda Index. However, alpha-ketoglutaric acid was not correlated with any of the other metabolites and may therefore reflect an independent pathway that may contribute to disturbances in glucose metabolism and insulin sensitivity. For example, alpha-ketoglutaric acid is a key intermediate in the citric acid cycle and is important for amino acid formation and the urea cycle. Alpha-ketoglutaric acid may be a marker of protein degradation and gluconeogenesis. Hepatic glutamate dehydrogenase catalyzes the reversible oxidative deamination of glutamate to α-ketoglutarate and ammonia, bridging amino acid-to-glucose pathways. In the current study, we observed higher levels of alpha-ketoglutaric acid to be associated with higher fasting glucose and HOMA-IR and a lower Matsuda Index in two independent cohorts of individuals without diabetes mellitus. Moreover, this metabolite was higher in individuals with diabetes mellitus. Since alpha-ketoglutaric acid was not associated with glycated hemoglobin, but only with measures of short-term insulin sensitivity, this metabolite may be more reflective of short-term glucose control instead of long-term glucose control.

A broad range of studies assessed the association between several lipid classes and type 2 diabetes mellitus(Mahendran et al. 2013a, b; Tillin et al. 2015; Wurtz et al. 2012, 2013; Mook-Kanamori et al. 2014; t Hart et al. 2018; Guasch-Ferre et al. 2016; Liu et al. 2017; Wang et al. 2017; Fall et al. 2016). Of these classes, for example, plasma phospholipids, triglycerides and sphingolipids were found to be associated with insulin resistance and type 2 diabetes mellitus onset (Mahendran et al. 2013a, b; Tillin et al. 2015; Wurtz et al. 2012, 2013; Mook-Kanamori et al. 2014; t Hart et al. 2018; Guasch-Ferre et al. 2016; Liu et al. 2017; Wang et al. 2017; Fall et al. 2016). In individuals with impaired fasting glucose and type 2 diabetes mellitus, higher levels of several saturated acids (e.g. palmitic and stearic) have been observed (Palomer et al. 2018; Guasch-Ferre et al. 2016). Our results are in agreement with these findings as we have identified palmitic (hexadecanoic acid) acid to be associated with higher fasting glucose levels.

We observed distinct metabolites to be associated with different measures of glucose metabolism. Of interest is that the metabolites that were associated with fasting insulin and HOMA-IR, which are based on fasting measures of glucose metabolism, were the same metabolites that associated with the Matsuda Index, which also takes into account postprandial measures of glucose metabolism. Since the metabolites that we found to be associated with fasting insulin, HOMA-IR and Matsuda Index were mainly overlapping, these measures may reflect similar biological pathways in the development of type 2 diabetes mellitus. Interestingly, only saturated fatty acids were associated with the Insulinogenic Index in the LLS, which is specific for β-cell function. We did not observe any association between glycated hemoglobin and any of the investigated metabolites. Glycated hemoglobin is the only index that we included that is able to assess glucose metabolism over a longer time period. Since none of the metabolites associated with glycated hemoglobin, the present observations may be more reflective of short-term insulin resistance and non-pathological, merely normal physiology.

The main strength of this study is the internal and external validation of our results in an independent cohort. The same associations were observed, thereby strengthening our results. The participants in the Leiden Longevity Study received a glucose drink and the participants of the NEO study received a mixed meal as a challenge, emphasizing the robustness of the observed associations. However, some limitations of our study have to be acknowledged. Because of the selection of individuals in the three batches (no random selection), we were not able to harmonize for batch effects using statistical techniques. Instead, we performed the analyses separately for the three batches using standardized metabolite data (mean = 0, s.d. = 1) and performed subsequent meta-analyses to combine the results. As a consequence, we minimized potential bias caused by the batch effects. In this study, we only observed higher levels of the identified metabolites to be associated with higher insulin resistance. High metabolite levels that are associated with lower insulin resistance might (1) have a smaller effect size that would require larger sample sizes, and (2) may only be measured on different platforms. Moreover, due to the observational nature of the study, we could not establish causality and address how the observed metabolites can affect insulin resistance or how insulin resistance may affect the metabolic profile, this will require dedicated prospective studies in the future.

Taken together, in a population of individuals without diabetes mellitus, we observed distinct metabolomic profiles to be associated with different measures of glucose metabolism. In total, we identified 12 metabolites to be associated with indices of glucose metabolism in individuals without diabetes mellitus. Most of these findings could be internally validated and externally replicated. Moreover, five of these metabolites associated with prevalent diabetes mellitus. Our results may improve the understanding of the mechanisms involved in disease etiology and thereby may contribute to improved diagnostics of the early metabolic disturbances preceding type 2 diabetes mellitus.

## Electronic supplementary material

Below is the link to the electronic supplementary material.

Supplementary file1 (XLSX 20 kb)

Supplementary file2 (XLSX 9 kb)
